# Feeder- and Drinker-Associated Microbiota Differ from Host-Associated Fecal Microbiota During a Commercial Broiler Production Cycle with Age-Associated Changes in Taxonomic Composition

**DOI:** 10.3390/ani16192987

**Published:** 2026-09-23

**Authors:** Jurgita Aksomaitiene, Aleksandr Novoslavskij, Beatrice Kasparaviciene, Snieguole Malakauskiene, Romualdas Sinkevicius, Loreta Serniene

**Affiliations:** 1 Department of Food Safety and Quality, Faculty of Veterinary Medicine, Lithuanian University of Health Sciences, Tilzes Str. 18, LT-47181 Kaunas, Lithuania; aleksandr.novoslavskij@lsmu.lt (A.N.); beatrice.kasparaviciene@lsmu.lt (B.K.); loreta.serniene@lsmu.lt (L.S.); 2 Department of Anatomy and Physiology, Faculty of Veterinary Medicine, Lithuanian University of Health Sciences, Tilzes Str. 18, LT-47181 Kaunas, Lithuania; snieguole.malakauskiene@lsmu.lt; 3 Department of Animal Nutrition, Faculty of Animal Sciences, Lithuanian University of Health Sciences, Tilzes Str. 18, LT-47181 Kaunas, Lithuania; romualdas.sinkevicius@lsmu.lt

**Keywords:** broiler, shotgun metagenomics, host–environment microbiota, fecal microbiota, taxonomic profiling

## Abstract

Broiler chickens are continuously exposed to microorganisms in the production environment, but the relationship between microorganisms on bird-contact surfaces and fecal microbiota remains unclear. In this exploratory observational study, shotgun metagenomic sequencing was used to compare microbial communities in feeder- and drinker-associated surface samples and individual fecal samples during one production cycle in a single commercial system housing Ross 308 broilers. The dataset comprised 22 individual fecal samples collected on days 1, 7, 19, 27, and 39 and four composite feeder- or drinker-associated environmental surface samples collected on days 1, 19, 27, and 39. Taxonomic composition, alpha and beta diversity, differential taxonomic signatures, and genus-level co-abundance patterns were evaluated. Environmental samples had greater richness and were dominated by Proteobacteria and Bacteroidetes, whereas host-associated fecal samples were dominated by Firmicutes and lactobacilli. Sample type explained 20.0% of variation in Bray–Curtis distances and 15.5% in Binary Jaccard distances (both *p* = 0.001). Within host-associated samples, Firmicutes increased from 51.4% on day 1 to 93.6% on day 39, while Proteobacteria decreased from 26.5% to 1.4%. Despite these taxonomic changes, host-associated communities did not form statistically distinct clusters by sampling day. Within the investigated production system, host-associated fecal microbial communities differed from those detected on the sampled feeder and drinker surfaces, while the relative abundance of dominant fecal taxa varied across sampling days. These findings are specific to the investigated production system and production cycle.

## Simple Summary

Microorganisms present in a poultry house are a potential source of exposure for broiler chickens, but the relationship between microorganisms on feeder and drinker surfaces and broiler fecal microbiota is not fully understood. We compared microbial communities in individual fecal samples from broilers with those in composite surface samples collected from feeders and drinkers during one commercial production cycle. The feeder- and drinker-associated samples had greater taxonomic richness and differed markedly from the communities associated with the birds. In broilers, the relative abundance of dominant bacterial groups differed across sampling days, with higher proportions of Firmicutes and lactobacilli and lower proportions of Proteobacteria later in the production cycle. However, the overall composition of the bird-associated communities did not separate into distinct groups according to sampling day. Fecal microbial profiles remained distinct from those detected on the sampled feeder and drinker surfaces. Because only one production system and one production cycle were examined, these findings describe the investigated dataset and should not be generalized to commercial broiler production.

## 1. Introduction

The gastrointestinal microbiota of broiler chickens is a complex and dynamic community whose composition varies across gut regions and changes throughout the production cycle [1,2]. These patterns reflect differences in physiology, substrate availability, transit time, and local conditions within the gastrointestinal tract [1]. In commercial poultry production, intestinal microbiota composition is associated with both host-related factors and external conditions, including feed, water systems, litter, dust, housing conditions, and other environmental reservoirs [3,4,5].

The production environment represents a continuous source of microbial exposure for broilers. Litter harbors diverse microbial communities and can influence gastrointestinal microbiota composition in broiler chickens [6]. Environmental management practices, including litter reuse and housing conditions, can further affect microbial exposure during production [7]. Exposure to environmental microorganisms is therefore only one part of broiler microbiota development. Conditions within the gastrointestinal tract, such as oxygen availability, nutrient gradients, host physiology, and immune activity, can affect which microorganisms become established in host-associated communities [8,9]. Collecting environmental and fecal samples during the same production cycle allows them to be compared under the same farm conditions. In this study, this comparison was used to determine whether the microbial composition of feeder and drinker surfaces differed from that of individual fecal samples. Age-related microbiota development has been repeatedly observed in broilers. Early communities are often enriched in facultative colonizers and Proteobacteria-related taxa, whereas later production stages are generally associated with increased dominance of Firmicutes, *Lactobacillus*, and other lactic acid bacteria [2,8,10,11,12,13]. Changes in dominant taxa may occur without statistically distinct whole-community clustering. Distinguishing age-associated changes in taxonomic composition from broader community-level separation helps clarify whether broiler microbiota maturation involves major restructuring of the community or primarily changes in the relative abundance of taxa already present.

Many poultry microbiome studies have used 16S rRNA amplicon sequencing, which is useful for broad community profiling but has limited taxonomic resolution, particularly at species level [3,5]. Shotgun metagenomic sequencing provides higher-resolution taxonomic information across multiple ranks and enables more detailed comparison of host-associated and environmental microbial communities. Recent poultry shotgun metagenomic studies have demonstrated the value of this approach for resolving microbial community composition in broiler-associated systems and poultry farm environments [14,15,16]. In this study, shotgun sequencing was used specifically for taxonomic profiling and community-structure analysis rather than functional characterization. Despite increasing interest in poultry microbiomes, several gaps remain in understanding host–environment microbiota dynamics during commercial broiler production. Many previous studies have focused either on intestinal or host-associated microbial communities alone, or on environmental reservoirs such as litter and housing-associated microbiota, whereas fewer studies have evaluated both environmental and host-associated components within the same production cycle. Few shotgun metagenomic studies have combined individual fecal samples with feeder- and drinker-associated surface samples within the same commercial production cycle. This study therefore provides an exploratory, descriptive comparison of these sample types within one production system and one production cycle. It also characterizes differences in the relative abundance of dominant fecal taxa across sampling days. These observations provide a reference for larger, independently replicated studies of broiler fecal microbiota and selected bird-contact surfaces.

The aim of this single-system observational study was to use shotgun metagenomic sequencing to profile taxonomic composition, alpha and beta diversity, differential taxonomic signatures, and genus-level co-abundance patterns in environmental and individual fecal samples collected within one defined commercial broiler production system during one production cycle. Specifically, we evaluated whether environmental and host-associated fecal microbial communities differed within the investigated production system, whether host-associated fecal microbiota differed across sampling days in the relative abundance of dominant taxa and in whole-community structure, and which genus-level co-abundance patterns were present across the pooled dataset.

## 2. Materials and Methods

### 2.1. Experimental Design and Sample Structure

The study was conducted during a single broiler production cycle within one commercial production system. Samples were collected at predefined time points throughout the production period, corresponding to days 1, 7, 19, 27, and 39. These sampling days were selected to represent early, intermediate, and late stages of the commercial production cycle and to describe differences in fecal microbial composition across sampling days.

The commercial farm and its management system have been described previously [17]. The present study involved a subsequent production cycle with a new broiler flock. Approximately 20,000 Ross 308 broilers were reared in the poultry house at a stocking density of approximately 19–20 birds/m^2^. The poultry house was closed and windowless, with no outdoor access. A four-phase feeding program was used: prestarter from placement to day 9–10, starter from day 9–10 to day 22–23, grower from day 22–23 to day 32–33, and finisher from day 32–33 until slaughter. Birds were floor-reared on peat-moss bedding and had ad libitum access to feed and drinking water. The farm operated an all-in-all-out management system, with thorough cleaning and disinfection performed before the start of each new production cycle. The final dataset included 26 metagenomic samples, comprising 22 individual fecal samples and 4 composite environmental surface samples. Freshly voided rectal feces (1 g per sample) were collected aseptically from individual broilers. Each bird was sampled only once, with different birds sampled at each production stage; samples were therefore treated as independent biological observations. The sex of the sampled birds was not determined.

At each environmental sampling point, 10 individual sterile swabs were collected from feeder or drinker surfaces and pooled to form one composite environmental sample. For each swab, an approximately 10 × 10 cm surface area was sampled using a standardized swabbing pattern, with the swab moved from top to bottom and then from one side to the other across the selected surface. The same sampling approach was applied at each environmental sampling point. The pooled swabs were placed in buffered peptone water (BPW; Liofilchem S.r.l., Roseto degli Abruzzi, Italy) for subsequent processing. All samples were transported at 4 °C and delivered to the laboratory within 2 h. Four composite environmental samples were obtained: one feeder-associated sample on day 1 and one drinker-associated sample on each of days 19, 27, and 39. Each composite sample therefore represented 10 individual surface swabs. These composite samples represented only the sampled feeder and drinker surfaces and were not considered representative of the broader poultry-house environment.

Because only one composite environmental sample was available at each sampled production stage, feeder- and drinker-associated samples were analyzed together as a single environmental category for the host–environment comparison. This grouping was used to provide a descriptive comparison between host-associated fecal microbiota and microbial communities detected on the sampled bird-contact surfaces, rather than to compare feeder- and drinker-specific microbial communities. Temporal changes in environmental microbiota were not evaluated because sampling was not replicated within individual production stages and the sampled surface type differed between day 1 and later time points. No environmental sample was available for day 7. Host-associated samples represented biological replication at the individual-bird level, with sample numbers as follows: day 1, *n* = 4; day 7, *n* = 3; day 19, *n* = 5; day 27, *n* = 5; and day 39, *n* = 5. Independent replication at the farm, poultry house, or production-cycle level was not performed.

### 2.2. DNA Extraction and Quality Assessment

Total microbial DNA was extracted from individual fecal samples and environmental samples using the Invitrogen PureLink Microbiome DNA Purification Kit (Thermo Fisher Scientific, Waltham, MA, USA) according to the manufacturer’s instructions. DNA purity was assessed using a NanoDrop spectrophotometer (Thermo Fisher Scientific, Waltham, MA, USA), and DNA concentration was quantified using the Qubit dsDNA HS Assay Kit (Thermo Fisher Scientific, Waltham, MA, USA). Extracted DNA was stored at −80 °C until library preparation and shotgun metagenomic sequencing.

### 2.3. Shotgun Metagenomic Sequencing and Quality Control

Shotgun metagenomic sequencing libraries were prepared following the standard Illumina workflow. Briefly, DNA samples passing quality assessment were fragmented, followed by end repair, A-tailing, adapter ligation, size selection, purification, and PCR amplification. Paired-end libraries with an average insert size of approximately 350 bp were sequenced on an Illumina NovaSeq X platform (Illumina, San Diego, CA, USA) using a 2 × 150 bp sequencing strategy. Raw sequencing reads were processed using fastp v0.23.2 [18] to remove adapter sequences and low-quality reads. Clean reads were aligned against the *Gallus gallus* reference genome using Bowtie2 v2.5.5 [19] and host-derived reads were removed before downstream microbial analysis. Across the 26 samples, sequencing generated 40.4–63.7 million clean reads per sample, corresponding to approximately 6.03–9.44 Gb of clean data. Sequencing quality was high, with Q20 values of 99.64–99.92% and Q30 values of 98.09–99.23%.

### 2.4. Metagenomic Assembly, Gene Prediction, and Taxonomic Profiling

Quality-controlled non-host reads were assembled de novo using MEGAHIT v1.2.9 [20] with default metagenomic assembly parameters. Metagenomic assembly generated 18,570–655,727 contigs per sample, with total assembly lengths ranging from approximately 27.4 to 977.7 Mb and N50 values ranging from 449 to 13,204 bp. Open reading frames were predicted from assembled contigs using MetaGeneMark v3.26 [21]. Predicted genes from all samples were clustered using CD-HIT v4.8.1 [22] to generate a non-redundant gene catalogue. Gene abundance was estimated by mapping quality-controlled reads back to the non-redundant gene catalogue and normalizing gene counts by gene length and sequencing depth. Taxonomic annotation was performed by aligning predicted protein sequences against the NCBI NR database using DIAMOND v2.0.15 [23]. Alignments were filtered using an e-value threshold of 1 × 10^−5^, and the best-supported database matches were used for downstream taxonomic assignment according to the annotation pipeline. Taxonomic abundance profiles were generated by assigning annotated genes to taxonomic ranks and summing normalized gene abundances within each taxon.

Taxonomic profiles were summarized at multiple ranks, including phylum, family, genus, and species levels. Relative abundance tables were generated and used for downstream diversity, differential abundance, and visualization analyses. Species-level assignments were interpreted as metagenomic taxonomic annotations rather than confirmed species identifications, particularly for taxa with potential clinical relevance.

### 2.5. Diversity, Differential Abundance, and Co-Abundance Network Analyses

Downstream diversity analyses, statistical testing, ordination, clustering, and visualization were performed using taxonomic abundance profiles generated by the bioinformatic workflow described above, with additional analyses conducted in R version 4.4.1 (R Foundation for Statistical Computing, Vienna, Austria). Alpha diversity was assessed using Shannon, Simpson, ACE, Chao1, and Pielou’s evenness indices. Diversity indices were compared between environmental and host-associated samples using Welch’s *t*-test because of unequal group sizes. For between-group alpha-diversity comparisons, Hedges’ g was additionally calculated as a standardized effect-size measure, and 95% confidence intervals were calculated for the mean differences between environmental and host-associated fecal samples. Differences among host-associated samples collected at different production stages were assessed using the Kruskal–Wallis test. Beta diversity was evaluated using Bray–Curtis dissimilarity and Binary Jaccard distance matrices calculated from presence/absence-transformed taxonomic profiles. Principal coordinate analysis (PCoA) was used to visualize differences in microbial community structure. Analysis of similarities (ANOSIM) and permutational multivariate analysis of variance (PERMANOVA) were used to test differences between environmental and host-associated samples and among host-associated sampling days. PERMANOVA was performed using permutation-based testing with 999 permutations. Homogeneity of multivariate dispersions was assessed using the betadisper procedure with spatial medians and bias adjustment for unequal group sizes, followed by permutation testing with 999 permutations. Welch’s *t*-test, the Kruskal–Wallis test, and permutation-based methods were selected because of the small and unequal group sizes and the limited ability to verify distributional assumptions.

Differential group-associated taxa were identified using linear discriminant analysis effect size (LEfSe) based on relative abundance data. Taxa with an absolute LDA score > 4.0 were retained for interpretation. Because the host-associated sampling-day groups contained only 3–5 samples, day-associated LEfSe results were treated as exploratory. These taxon-level signatures were not considered evidence of statistically significant whole-community separation among production stages.

Genus-level differences between environmental and host-associated samples were further evaluated using the Wilcoxon rank-sum test. The analysis pipeline additionally calculated false discovery rate (FDR)-adjusted *p*-values to account for multiple testing. FDR-adjusted *p* < 0.05 was considered statistically significant after correction for multiple testing, whereas findings based only on nominal *p* < 0.05 were treated as exploratory. Genera with nominal *p* < 0.05 were visualized using hierarchical clustering heatmaps based on standardized relative abundance values. Hierarchical clustering was performed using distance-based clustering of standardized genus-level abundance profiles. An exploratory genus-level co-abundance network was constructed using relative abundance data from dominant bacterial genera to visualize broad co-abundance patterns across the pooled dataset. Spearman’s rank correlation analysis was used to identify co-abundance patterns among genera. Two-sided *p*-values were adjusted for multiple comparisons using the Benjamini–Hochberg false discovery rate (FDR) procedure. For network visualization and topology analysis, the 100 strongest positive Spearman correlations were retained; all retained associations remained significant after FDR correction (adjusted *p* < 0.05). This cutoff was used to retain the strongest associations while keeping the network interpretable. It was not intended to represent a biologically defined significance threshold, and the choice of this cutoff may influence calculated network topology. Network topology parameters, including node degree, clustering coefficient, density, modularity, and module structure, were calculated from the retained correlation network. Because the network was based on relative abundance correlations, network edges were interpreted as co-abundance patterns rather than direct ecological interactions.

## 3. Results

### 3.1. Taxonomic Composition

Metagenomic sequencing identified diverse microbial communities in environmental and host-associated fecal samples collected during the broiler production cycle. Across all samples, taxonomic profiling yielded 7 kingdoms, 169 phyla, 166 classes, 339 orders, 787 families, 3229 genera, and 18,767 species-level taxonomic annotations.

Differences in community composition were observed between environmental and host-associated fecal microbiota at multiple taxonomic levels (Figure 1). At the phylum level, environmental samples were dominated by Proteobacteria (65.1%) and Bacteroidetes (19.0%), whereas host-associated samples were characterized by a predominance of Firmicutes (74.5%). Proteobacteria accounted for 13.4% of the host-associated fecal microbiota, while Actinobacteria were detected at similar relative abundances in environmental (4.1%) and host-associated (3.8%) samples. At the genus level, the most abundant genera in environmental samples were *Acinetobacter* (11.1%), *Paracoccus* (6.4%), *Klebsiella* (5.9%), *Acidovorax* (3.7%), and *Pseudomonas* (3.1%). Other detected genera included *Epilithonimonas*, *Brevundimonas*, *Sphingobacterium*, and *Chryseobacterium*. In contrast, host-associated fecal microbiota were dominated by *Lactobacillus* (35.7%), followed by *Ligilactobacillus* (12.7%), *Escherichia* (10.4%), and *Limosilactobacillus* (5.1%). Other frequently detected genera included *Chlamydia* (3.8%), *Clostridium* (3.7%), *Streptococcus*, *Bacteroides*, and *Enterococcus* (Figure 2). Within host-associated fecal samples, the relative abundance of dominant phyla varied across sampling days. The relative abundance of Firmicutes increased progressively from 51.4% on day 1 to 57.6% on day 7, 66.6% on day 19, 92.1% on day 27, and 93.6% on day 39. In contrast, Proteobacteria declined from 26.5% on day 1 to 1.4% by day 39. Actinobacteria reached their highest abundance on day 19 (11.8%), whereas Bacteroidetes remained at relatively low abundances throughout the production period (0.1–8.7%).

At the genus level, *Lactobacillus* increased from 20.4% on day 1 to 64.8% on day 27 and remained highly abundant on day 39 (42.8%). *Ligilactobacillus* showed a marked increase during later stages of production, reaching 29.9% on day 39. Conversely, *Escherichia* decreased steadily from 20.5% on day 1 to 0.8% on day 39. *Limosilactobacillus* became more abundant after day 27, while *Enterococcus* exhibited a transient peak on day 19 (6.4%).

At the class level, Bacilli were more abundant at later sampling days, whereas Gammaproteobacteria, Clostridia, and Actinomycetia were detected at higher proportions during the early production stages. Similar differences across sampling days were also observed at lower taxonomic ranks.

At the family level, Enterobacteriaceae, Oscillospiraceae, Clostridiaceae, and Lachnospiraceae predominated during the first week, whereas Lactobacillaceae showed higher relative abundance at later sampling days and was one of the dominant bacterial families in samples collected from older birds.

Differential abundance analysis was then used to examine these differences at the genus level. Exploratory Wilcoxon rank-sum analysis identified 1247 genera with nominal *p* < 0.05 between environmental and host-associated samples. Of these, 244 remained statistically significant after Benjamini–Hochberg FDR correction (adjusted *p* < 0.05). Environmental samples showed higher relative abundances of *Epilithonimonas*, *Emticicia*, *Methylobacillus*, *Kaistella*, *Empedobacter*, *Pseudorhodoferax*, and *Polaromonas*, whereas host-associated samples showed higher relative abundances of *Agathobaculum*, *Intestinimonas*, *Massilimicrobiota*, *Pediococcus*, and *Liquorilactobacillus*.

### 3.2. Alpha Diversity

Alpha-diversity indices were generally higher in environmental than in host-associated fecal samples, although statistical significance differed among indices. Mean Shannon diversity reached 5.34 in environmental samples, compared with 3.19 in host-associated samples. Similarly, richness estimators were substantially higher in environmental samples, with mean ACE and Chao1 values of 7756.7 and 7748.3, respectively, whereas host-associated samples showed mean values of 2232.2 and 2226.5. Environmental samples also had higher Simpson diversity (0.966) and Pielou’s evenness (0.597) than host-associated samples (0.825 and 0.421, respectively). Differences between environmental and host-associated fecal samples were detected for ACE (*p* = 0.046; Hedges’ g = 2.68; mean difference = 5524.5, 95% CI: 185.3–10,863.7), Chao1 (*p* = 0.046; Hedges’ g = 2.68; mean difference = 5521.7, 95% CI: 189.5–10,853.9), and Simpson diversity (*p* = 0.0043; Hedges’ g = 0.90; mean difference = 0.141, 95% CI: 0.051–0.231). Shannon diversity (*p* = 0.056; Hedges’ g = 1.56; mean difference = 2.146, 95% CI: −0.096–4.388) and Pielou’s evenness (*p* = 0.073; Hedges’ g = 1.29; mean difference = 0.176, 95% CI: −0.026–0.378) were also higher in environmental samples, although their 95% confidence intervals for the mean difference included zero. Differences in Shannon diversity between sample types and production stages are shown in Figure 3. Within host-associated samples, alpha diversity varied numerically throughout the production cycle. The highest mean Shannon diversity was observed on day 19 (3.89), together with the highest mean ACE richness value (3483.3) and the highest Pielou’s evenness values. Lower diversity and richness values were observed on days 1 and 7, whereas samples collected on days 27 and 39 showed broader variation across diversity indices, indicating greater variability among individual samples. However, differences in alpha diversity among host-associated sampling days were not statistically significant.

### 3.3. Beta Diversity

Beta diversity was evaluated to compare microbial community composition between environmental and host-associated fecal samples and among host-associated sampling days. A clear separation between environmental and host-associated fecal microbial communities was observed. Analysis of similarities (ANOSIM) based on Bray–Curtis dissimilarities demonstrated a strong difference between sample types (R = 0.924, *p* = 0.001). Similar results were obtained using Binary Jaccard distances, which also showed significant separation between environmental and host-associated samples (R = 0.700, *p* = 0.001). PERMANOVA showed a significant effect of sample type on community composition. Based on Bray–Curtis distances, sample type explained 20.0% of the variation in microbial community composition (R^2^ = 0.200, *p* = 0.001). Using Binary Jaccard distances, sample type explained 15.5% of the total variation (R^2^ = 0.155, *p* = 0.001). Bias-adjusted tests of multivariate dispersion showed no significant difference in dispersion between environmental and host-associated samples for either Bray–Curtis (F = 0.118, *p* = 0.73) or Binary Jaccard distances (F = 2.859, *p* = 0.09). PCoA showed distinct clustering of environmental and host-associated samples with minimal overlap between groups (Figure 4). For Bray–Curtis distances, the first two principal coordinates explained 42.3% of the total variation (PC1 = 25.73%, PC2 = 16.58%). For Binary Jaccard distances, the first two principal coordinates explained 41.9% of the variation (PC1 = 22.82%, PC2 = 19.04%). These results describe separation within the present dataset and should be interpreted in the context of the limited environmental sample size (*n* = 4).

For host-associated sampling days, ANOSIM analysis based on Bray–Curtis distances yielded a low R value (R = 0.065, *p* = 0.139), while Binary Jaccard analysis produced a similar result (R = 0.082, *p* = 0.123). PERMANOVA analysis also showed no significant differences among sampling days, with Bray–Curtis distances explaining 20.0% of the variation (R^2^ = 0.200, *p* = 0.126) and Binary Jaccard distances explaining 19.0% of the variation (R^2^ = 0.190, *p* = 0.138). PCoA of host-associated fecal samples showed substantial overlap among production stages, with no distinct clustering by sampling day.

### 3.4. Differential Taxonomic Signatures

Linear discriminant analysis effect size (LEfSe) was used to identify taxa associated with environmental and host-associated fecal microbial communities, as well as taxa associated with different sampling days. LEfSe identified distinct taxonomic signatures associated with environmental and host-associated fecal samples. Host-associated samples were enriched with Firmicutes-associated taxa, including Bacilli, Lactobacillales, Lactobacillaceae, *Lactobacillus*, *Ligilactobacillus*, and *Limosilactobacillus*. The highest LDA scores in host-associated samples were observed for Firmicutes, Bacilli, Lactobacillales, Lactobacillaceae, and *Lactobacillus*. Species-level taxa reported below represent metagenomic taxonomic assignments based on the reference database and should not be interpreted as culture-confirmed species detections. Several species-level taxa were also associated with host-associated samples, including *Lactobacillus crispatus*, *Lactobacillus johnsonii*, *Lactobacillus helveticus*, *Ligilactobacillus salivarius*, *Ligilactobacillus aviarius*, and *Limosilactobacillus reuteri*. Additional host-associated taxonomic signatures included Clostridia and Eubacteriales (Figure 5). Environmental samples were primarily characterized by Proteobacteria-associated taxa. The main taxonomic signatures enriched in environmental samples included Proteobacteria, Alphaproteobacteria, Betaproteobacteria, Burkholderiales, Comamonadaceae, Moraxellales, Moraxellaceae, *Acinetobacter*, and *Acinetobacter baumannii*. Other environmental taxonomic signatures included *Paracoccus*, Rhodobacteraceae, Rhodobacterales, Flavobacteriia, Flavobacteriales, Weeksellaceae, *Epilithonimonas*, Sphingobacteriia, and Sphingobacteriales. These taxa had absolute LDA scores >4.0 and were assigned to the environmental group. Exploratory LEfSe analysis of host-associated fecal samples identified several day-associated taxonomic signatures. Day 1 samples were characterized by Enterobacterales. Day 7 samples were associated with *Escherichia*, *Escherichia coli*, *Enterococcus*, *Enterococcus faecium*, Enterococcaceae, *Nocardia*, and Nocardiaceae. Day 19 samples were characterized by Micrococcales, Dermabacteraceae, *Brachybacterium*, and Staphylococcaceae. Day 27 samples were associated with *Limosilactobacillus oris*. No taxa with LDA scores above the selected threshold were identified for day 39. Because the sampling-day groups were small (*n* = 3–5) and no significant whole-community separation was detected in the ANOSIM or PERMANOVA analyses, these day-associated signatures were treated as hypothesis-generating and were not interpreted as reproducible age-specific taxonomic patterns. Genera showing nominal differences between groups (Wilcoxon rank-sum test, *p* < 0.05) were visualized by hierarchical clustering, which separated environmental and host-associated fecal samples based on relative abundance profiles (Figure 6).

### 3.5. Genus-Level Co-Abundance Network Analysis

An exploratory genus-level co-abundance network was constructed to visualize associations among dominant bacterial genera across the pooled dataset. The final network comprised 41 genera connected by the 100 strongest retained positive correlations. Spearman correlation coefficients ranged from 0.900 to 0.974, and all retained associations remained significant after FDR correction (adjusted *p* < 0.05). The network had an average node degree of 4.88, a clustering coefficient of 0.759, a density of 0.122, and a modularity of 0.421 (Figure 7).

The retained positive-correlation network consisted of a large central cluster together with several smaller peripheral subnetworks. The central cluster was composed primarily of anaerobic genera, including *Drancourtella*, *Enterocloster*, *Flavonifractor*, *Subdoligranulum*, *Agathobaculum*, *Butyricicoccus*, *Faecalibacterium*, *Blautia*, and *Gemmiger*, which were connected by multiple retained co-abundance links. Several genera exhibited particularly high network connectivity. *Drancourtella, Enterocloster*, and *Flavonifractor* each showed 13 retained co-abundance links, representing the highest degree values within the network. *Agathobaculum* and *Butyricicoccus* were connected to 12 other genera, while *Merdimonas*, *Anaeromassilibacillus*, *Subdoligranulum*, and *Intestinibacillus* each showed 10 retained co-abundance links.

In contrast, several genera occupied peripheral positions within the network and formed smaller peripheral clusters. These included *Paracoccus*, *Pedobacter*, *Chryseobacterium, Brevundimonas, Sphingomonas*, *Delftia*, *Corynebacterium*, *Mammaliicoccus*, *Salmonella*, *Klebsiella*, *Lactobacillus*, and *Limosilactobacillus*. *Lactobacillus* had the highest mean relative abundance and therefore appeared as the largest node, despite having relatively few retained connections. Because the network was based on relative abundance correlations, the observed edges should be interpreted as co-abundance patterns rather than direct ecological interactions.

## 4. Discussion

This study compared composite feeder- and drinker-associated surface samples with individual fecal samples collected during one commercial broiler production cycle. The sample types differed in taxonomic composition and alpha and beta diversity, while LEfSe and hierarchical clustering identified additional sample-type-associated patterns. Given the limited environmental sampling and unequal sample sizes between groups, these differences should be interpreted as specific to the investigated production system and production cycle.

Environmental samples showed higher richness and were dominated by Proteobacteria and Bacteroidetes, whereas host-associated samples were characterized by Firmicutes and lactobacilli. Sample type explained 20.0% of variation in Bray–Curtis distances and 15.5% in Binary Jaccard distances. Similar differentiation between poultry house environments and host-associated microbiota has been reported in studies of litter, housing environments, intestinal microbiota, and host-associated microbial communities [1,4,6,24,25].

Feeder and drinker surfaces were selected because birds come into direct contact with them during feeding and drinking. These samples represented selected bird-contact surfaces rather than the entire poultry-house environment. Because the environmental category combined one feeder-associated sample with three drinker-associated samples, surface-specific differences may have contributed to variation within this group.

Within the present dataset, host-associated fecal communities were compositionally distinct from those detected on the sampled feeder and drinker surfaces. Host physiology, nutrient availability, oxygen gradients, immune responses, and microbial competition may contribute to differences between environmental and host-associated microbial communities [8,9,26]. Their contribution could not be assessed because microbial transmission and host selection mechanisms were not examined. Differences across sampling days in host-associated fecal microbiota were more apparent in the relative abundance of dominant taxa than in whole-community structure. Firmicutes increased from 51.4% on day 1 to 93.6% on day 39, whereas Proteobacteria declined from 26.5% to 1.4%. At the genus level, *Lactobacillus* increased from 20.4% on day 1 to 64.8% on day 27 and remained abundant on day 39, while *Escherichia* declined from 20.5% to 0.8%.

Age-associated changes in broiler microbiota have been linked to intestinal development and immune function [1,3,27], as well as to nutrient utilization and productive performance [1,3]. Previous studies have also shown marked age-related changes in microbial composition during broiler growth, including increasing dominance of Firmicutes and lactobacilli and changes in Proteobacteria-associated taxa [8,10]. Cecal microbiota development has additionally been associated with changes in nutrient metabolism [13]. In the present study, samples did not form statistically distinct clusters by sampling day in ANOSIM, PERMANOVA, or PCoA analyses. Relative abundance profiles showed differences in dominant taxa across sampling days, whereas beta-diversity analyses indicated substantial overlap in overall community structure. This combination of taxonomic differences and community-level overlap does not demonstrate a complete restructuring of the overall microbial community across the production cycle. Given the small sampling-day groups, these taxonomic patterns should be regarded as hypothesis-generating. LEfSe, differential abundance analysis, and hierarchical clustering identified additional taxonomic differences between sample types. Host-associated taxonomic signatures were mainly related to *Firmicutes*, *Bacilli*, *Lactobacillales*, *Lactobacillaceae*, *Lactobacillus*, *Ligilactobacillus*, and *Limosilactobacillus*. Environmental signatures were mainly associated with Proteobacteria-related groups, including *Acinetobacter*, *Paracoccus, Moraxellaceae*, *Comamonadaceae*, and *Burkholderiales*. Similar differences between environmental and host-associated communities have been reported in poultry litter–gut studies, where environmental and host-associated communities remained differentiated despite continuous microbial exchange [24,25]. Ordination, LEfSe, and heatmap analyses showed broadly consistent differences between sample types, although not all genera identified by nominal Wilcoxon testing remained significant after FDR correction. Day-associated LEfSe signatures were treated as hypothesis-generating because of the small group sizes and the lack of significant whole-community separation among sampling days. Shotgun metagenomic sequencing provided taxonomic profiles across multiple ranks and allowed a more detailed comparison of environmental and host-associated fecal communities than 16S rRNA amplicon sequencing. More than 18,000 species-level taxonomic annotations were assigned; however, this number reflects database-based assignments and should not be interpreted as confirmed species richness. This is particularly relevant for clinically important or regulated taxa, including species-level assignments within *Acinetobacter, Escherichia*, *Salmonella*, *Klebsiella*, and *Enterococcus*. Species-level assignments should be treated as taxonomic annotations, not confirmed pathogen detections, unless supported by culture, targeted read mapping, or genome-resolved analysis. Recent poultry metagenomic studies have similarly shown that shotgun sequencing improves taxonomic resolution and supports downstream functional profiling [14,15,28].

Proteobacteria-associated groups, including *Acinetobacter*, *Moraxellaceae*, *Comamonadaceae*, and *Burkholderiales*, were enriched in environmental samples, but their detection does not by itself indicate antimicrobial resistance or pathogenicity. Determining whether these taxa carry antimicrobial resistance determinants, virulence-associated traits, or persistence-related functions will require targeted functional analysis. The genus-level co-abundance network was used as an exploratory visualization of co-abundance patterns across the pooled dataset. Several anaerobic genera formed a central cluster, whereas *Lactobacillus*, despite its high relative abundance, showed limited connectivity. In this dataset, abundance and network centrality were not equivalent, and greater connectivity was not interpreted as evidence of ecological importance. Correlation-based networks do not demonstrate direct microbial interactions and are sensitive to compositional effects, shared habitat preferences, and differences between sample types [29,30,31,32]. Because host-associated and environmental samples were included in the pooled network, some correlations may partly reflect habitat-related differences in taxon abundance rather than within-habitat ecological associations. The 100 strongest positive correlations were retained for network visualization. This threshold was not biologically defined, and the resulting network topology may vary depending on the number of correlations retained.

Several limitations should be considered. The study was conducted within a single commercial production system during one broiler production cycle. Although host-associated samples represented biological replication at the individual-sample level, independent replication across farms, poultry houses, or repeated production cycles was not performed. The observed patterns are specific to the investigated production system and production cycle and should not be generalized to commercial broiler production. The small and unequal sample sizes between the environmental (*n* = 4) and host-associated fecal (*n* = 22) groups also reduced the precision of between-group alpha-diversity estimates. The small host-associated sampling-day groups (*n* = 3–5) also limited the ability to detect differences among sampling days.

Environmental sampling comprised four composite feeder- or drinker-associated surface samples, each generated from 10 individual swabs. This composite design increased the surface coverage represented by each sample but did not provide independent environmental replication within individual production stages.

In addition, the environmental category included both feeder- and drinker-associated samples, which may have introduced surface-specific heterogeneity because these two environmental compartments were not independently replicated or compared. Other potentially important poultry-house reservoirs, including litter, dust, feed, drinking water, air, and additional surfaces, were not sampled. The environmental component therefore represents selected bird-contact surfaces rather than the entire poultry-house microbial environment, and temporal changes in environmental microbiota could not be evaluated.

The analyses were based on relative abundance data, which may influence differential abundance and co-abundance patterns. Changes in relative abundance cannot be interpreted as changes in absolute microbial load. Day-associated LEfSe signatures were treated as hypothesis-generating, and species-level assignments require targeted validation before pathogen-specific conclusions can be drawn. In addition, taxonomic profiles were dependent on the reference database, classification algorithm, and filtering thresholds used for taxonomic assignment. This study focused on taxonomic composition and community structure. Functional profiling of antimicrobial resistance genes, virulence-associated traits, and metabolic pathways represents a separate analytical step beyond the scope of this study. The dataset supports taxonomic and community-structure comparisons but does not establish microbial transmission, causal host–environment relationships, or functional consequences.

## 5. Conclusions

This exploratory study provides shotgun metagenomic taxonomic profiles of composite feeder- and drinker-associated environmental surface samples and host-associated fecal samples within a single commercial broiler production system during one production cycle. Within the investigated system, feeder- and drinker-associated surface samples and host-associated fecal samples differed in taxonomic composition, but causal relationships between the environmental and fecal microbial communities were not assessed. Within host-associated fecal samples, the relative abundance of dominant taxa varied across sampling days, with higher proportions of Firmicutes and lactobacilli and lower proportions of Proteobacteria at later sampling points. Production stage was not associated with statistically distinct whole-community clustering among host-associated fecal samples.

Because the study was limited to one production system and one production cycle, broader multi-farm and multi-cycle studies with expanded environmental sampling and functional analyses are needed to assess the generalizability of these observations.

## Figures and Tables

**Figure 1:**
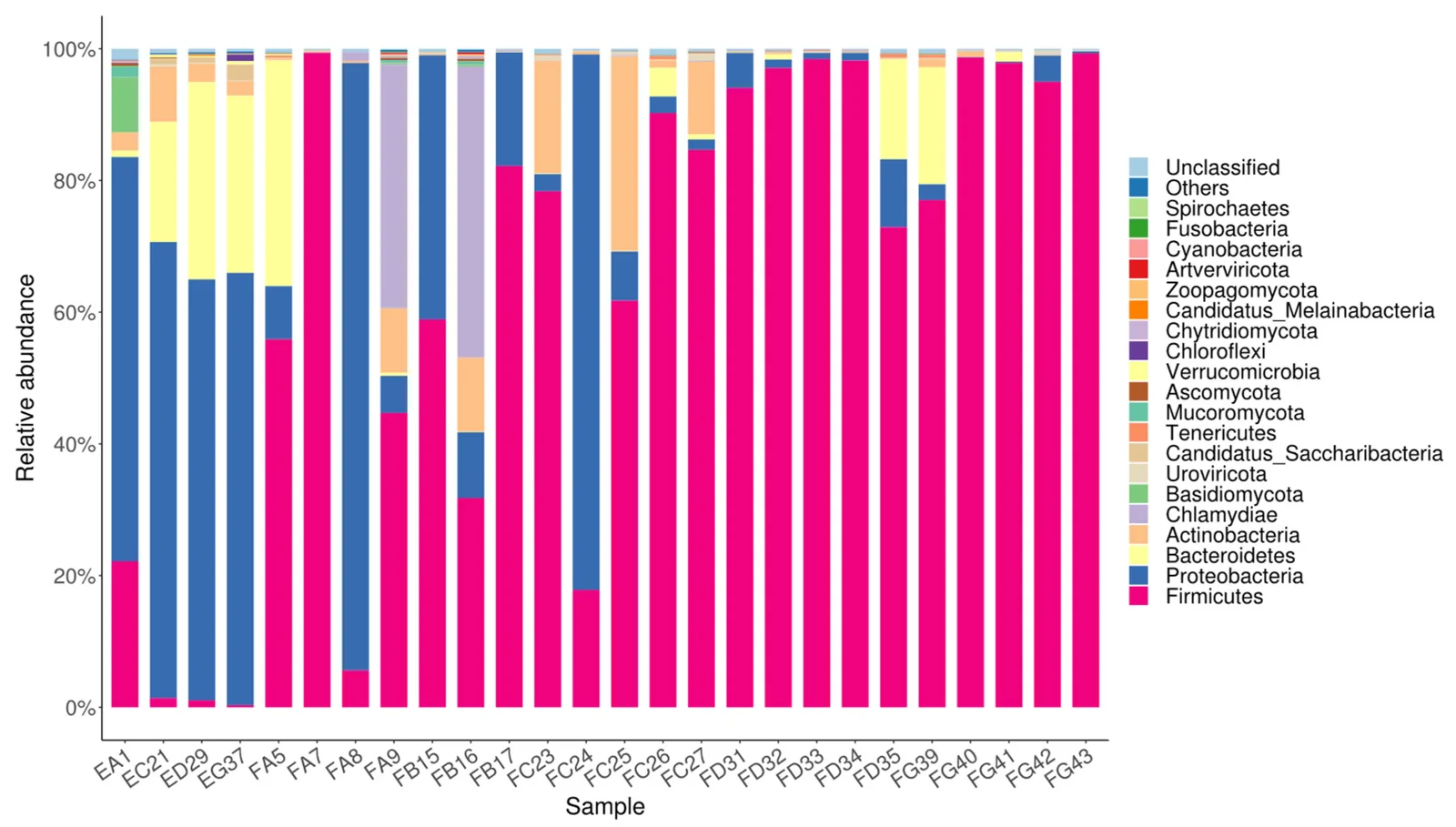
Relative abundance of dominant bacterial phyla in environmental samples (E) and host-associated fecal samples (F) collected at different sampling points. Each bar represents an individual sample.

**Figure 2:**
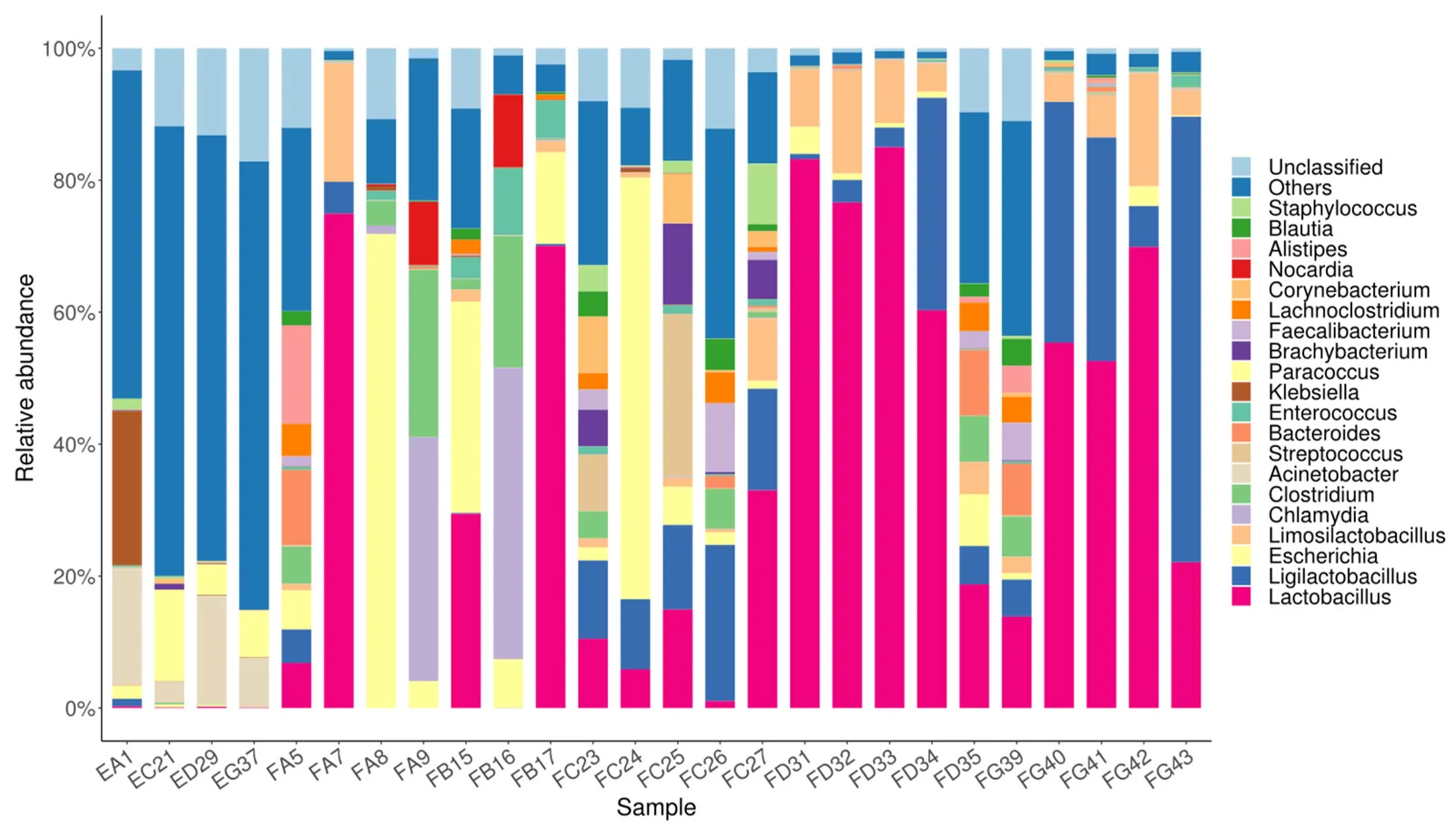
Relative abundance of dominant bacterial genera in environmental samples (E) and host-associated fecal samples (F) collected at different sampling points. Each bar represents an individual sample.

**Figure 3:**
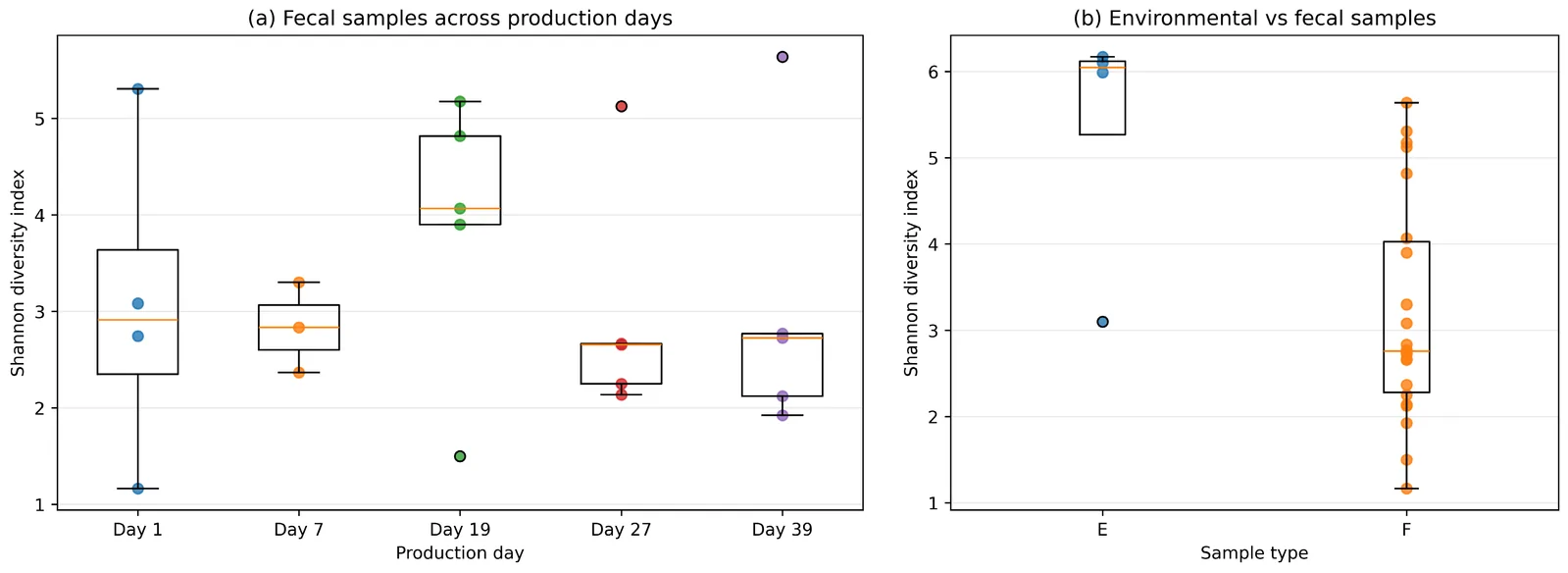
Shannon diversity index comparison across host-associated sampling days and between sample types. (**a**) Shannon diversity variation among host-associated samples collected on days 1, 7, 19, 27, and 39. (**b**) Comparison of Shannon diversity between environmental (E) and host-associated (F) samples, showing numerically higher Shannon diversity values in environmental microbiota. Colored dots represent individual samples; in panel (**a**), colors indicate sampling day, and in panel (**b**), blue and orange indicate environmental and host-associated fecal samples, respectively. Orange horizontal lines within the boxes indicate median values.

**Figure 4:**
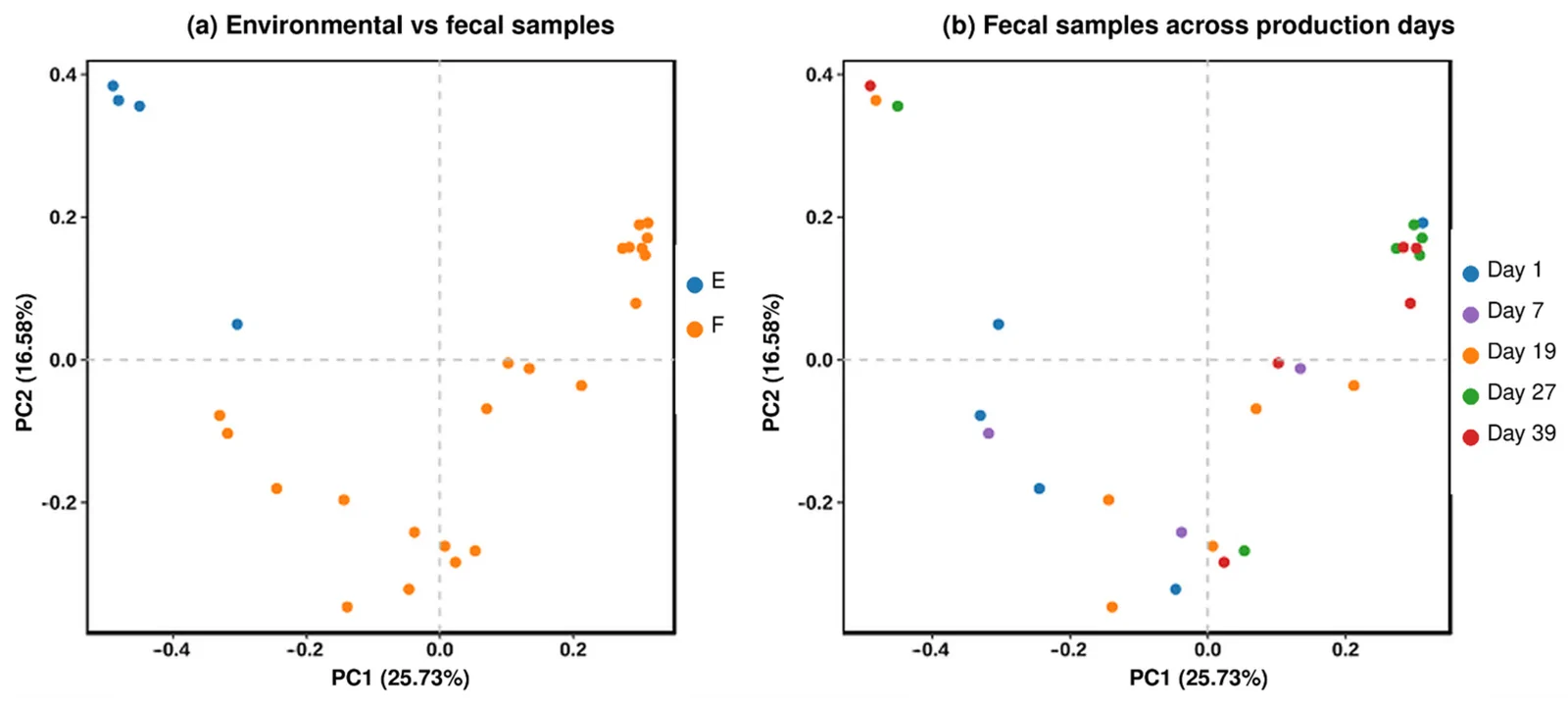
Principal coordinate analysis (PCoA) based on Bray–Curtis dissimilarities. (**a**) Ordination of environmental (E) and host-associated (F) samples showing clear separation between sample types (PC1 = 25.73%, PC2 = 16.58%). (**b**) Ordination of host-associated samples collected on days 1, 7, 19, 27, and 39 showing substantial overlap among production stages.

**Figure 5:**
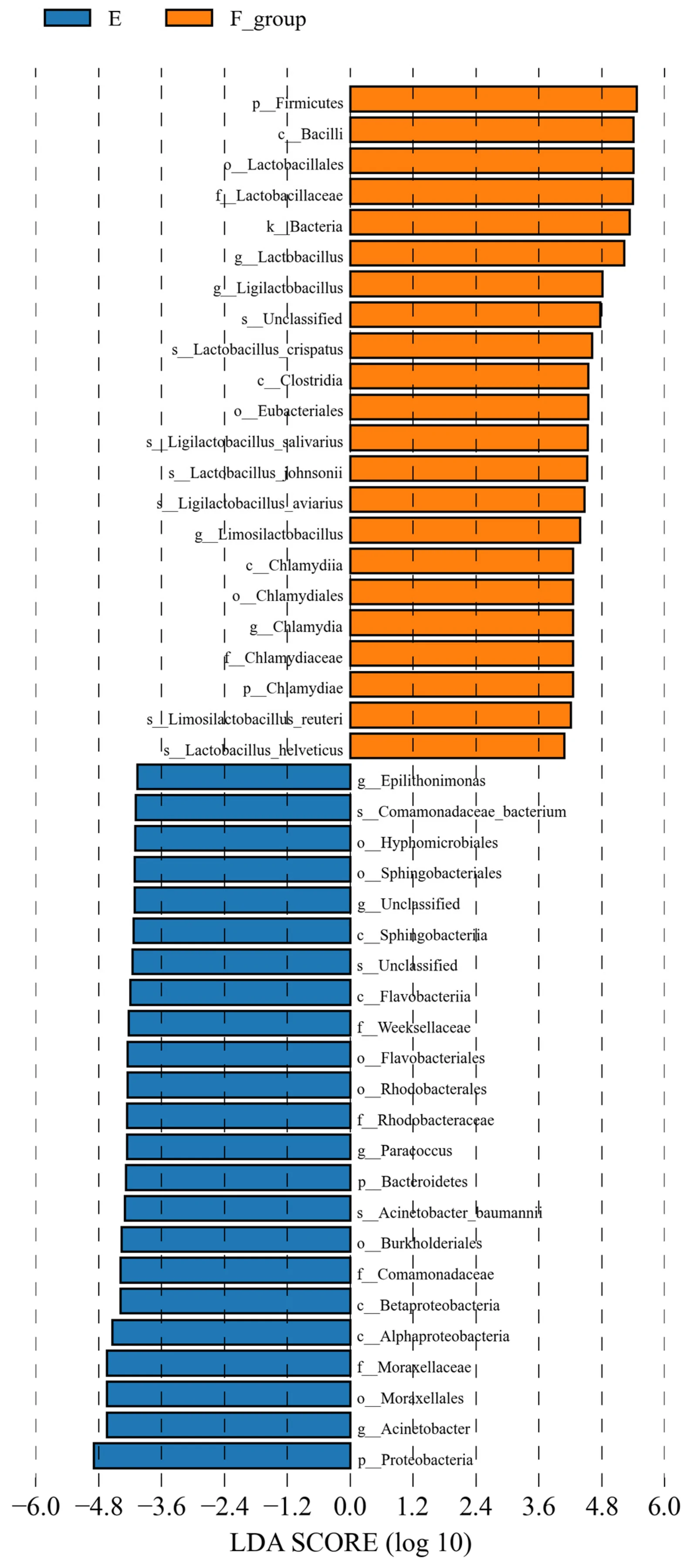
Linear discriminant analysis effect size (LEfSe) plot showing differential taxonomic signatures between environmental (E) and host-associated fecal (F_group) samples. Only taxonomic signatures with an absolute linear discriminant analysis (LDA) score > 4.0 are displayed.

**Figure 6:**
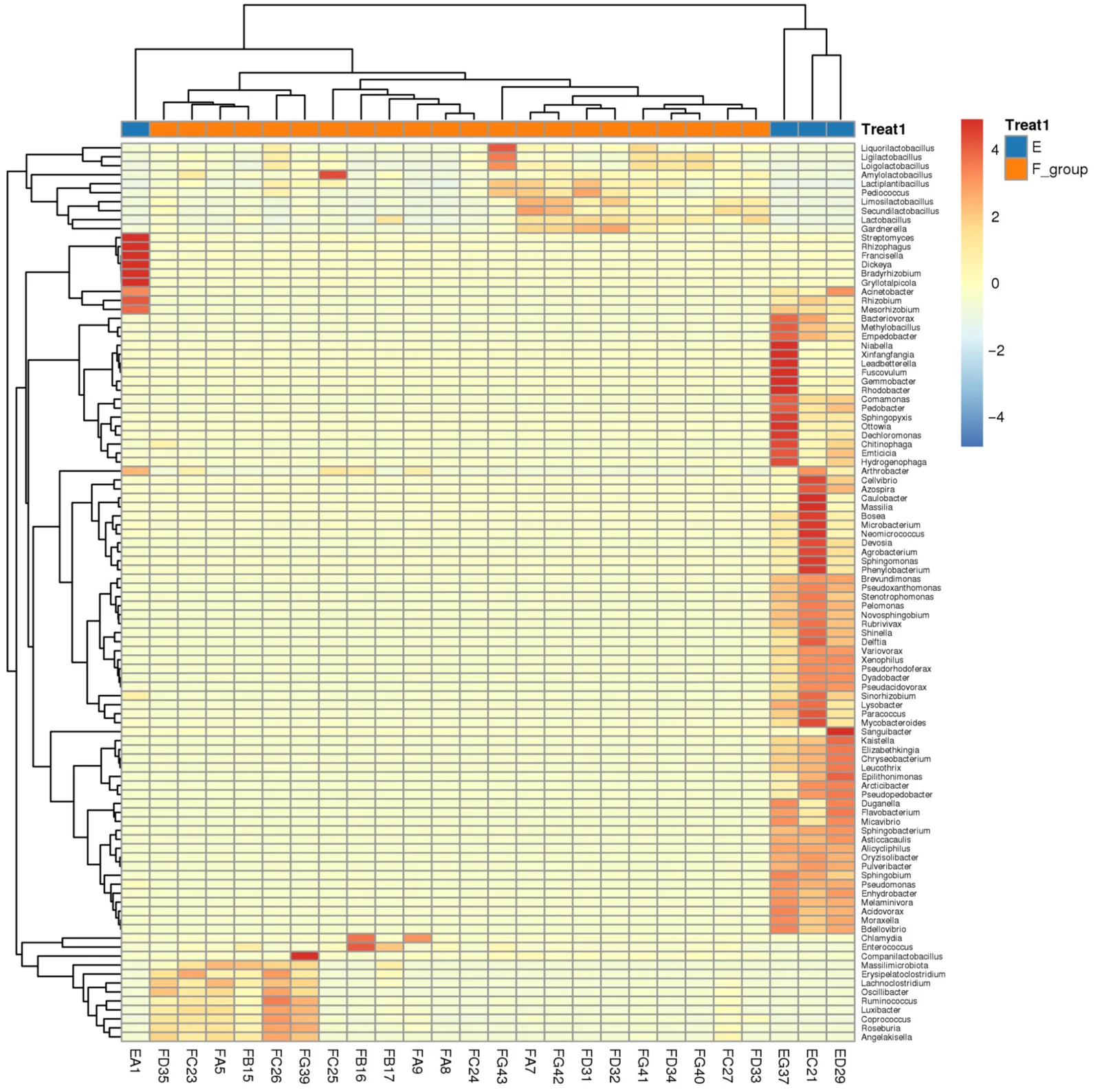
Differential abundance heatmap of bacterial genera showing differences between environmental (E) and host-associated (F_group) samples in the Wilcoxon rank-sum analysis (nominal *p* < 0.05). Rows represent bacterial genera and columns represent individual samples. Color intensity indicates standardized relative abundance values, with warmer colors corresponding to higher relative abundance and cooler colors corresponding to lower relative abundance. The annotation bar indicates sample type: E, environmental samples; F_group, host-associated fecal samples. Both samples and genera were hierarchically clustered based on abundance profiles. The heatmap is exploratory and includes genera selected using nominal *p* < 0.05; not all displayed genera remained significant after FDR correction.

**Figure 7:**
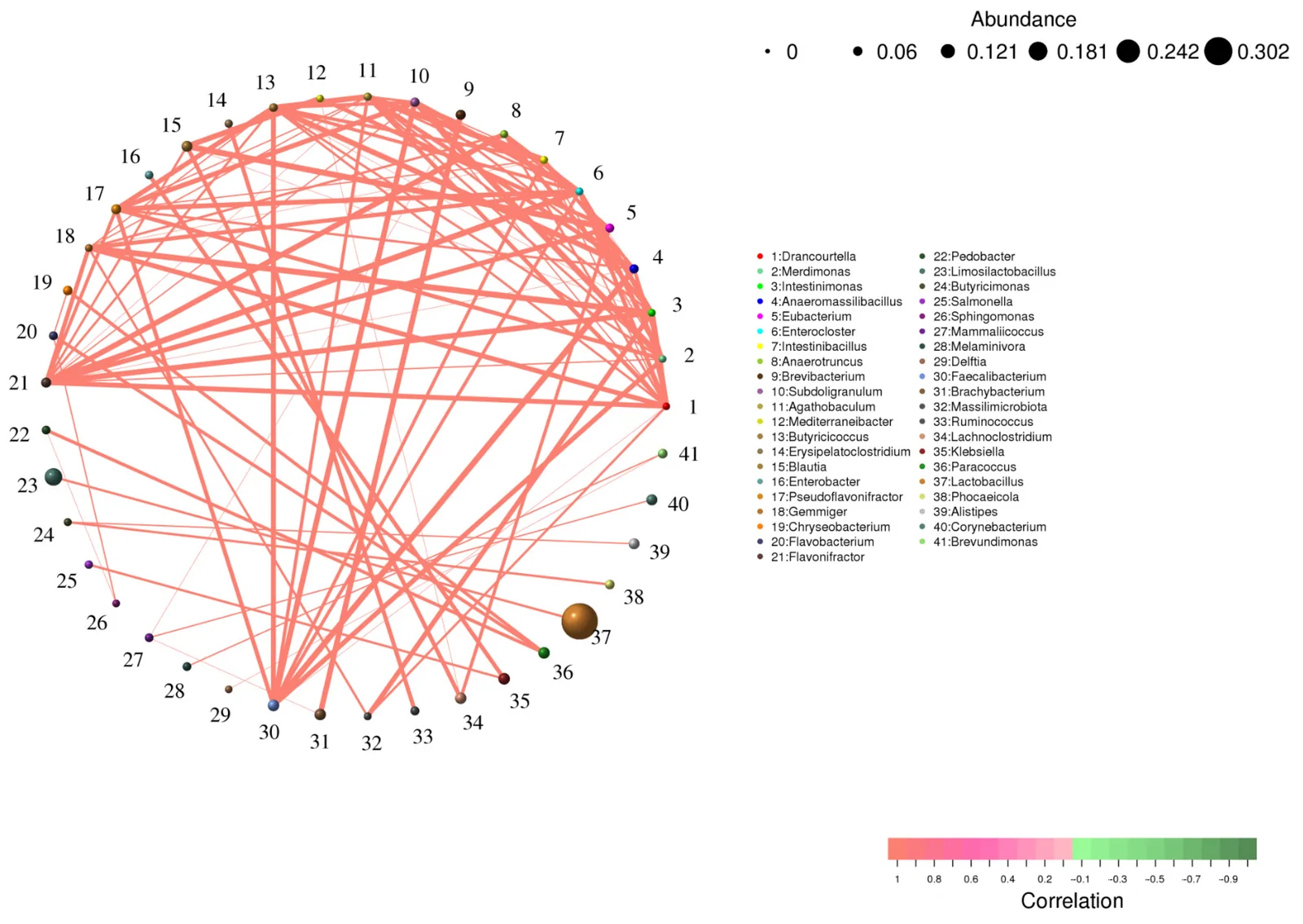
Genus-level co-abundance network based on relative abundance data. Nodes represent bacterial genera and edges represent the 100 strongest retained positive correlations. Node size corresponds to mean relative abundance. Edges represent co-abundance correlations and should not be interpreted as direct microbial interactions. Edge width is proportional to the Spearman correlation coefficient, with thicker lines indicating stronger correlations.

## Data Availability

The shotgun metagenomic sequencing data have been deposited in the NCBI Sequence Read Archive (SRA) under BioProject accession number PRJNA1520518. Processed data supporting the analyses presented in this study are available from the corresponding author upon reasonable request.
